# Description of Encephalon Morphology of Nile Tilapia *Oreochromis niloticus* (Linnaeus, 1758) (Perciformes: Cichlidae) From Brazil

**DOI:** 10.1111/ahe.70082

**Published:** 2026-01-07

**Authors:** Anna Luiza de Souza Pereira, Echily Sartori, Gabriela Munis Campos, Carolina Demétrio Ferreira, Pedro Pierro Mendonça, Cristiane dos Santos Vergilio

**Affiliations:** ^1^ Laboratório de Morfologia Animal, Departamento de Biologia, Centro de Ciências Exatas, Naturais e da Saúde Universidade Federal do Espírito Santo ‐ Campus Alegre Alegre Espírito Santo Brazil; ^2^ Laboratório de Nutrição e Produção de Peixes Ornamentais Instituto Federal do Espírito Santo – Campus Alegre Alegre Espírito Santo Brazil

**Keywords:** anatomy, brain, histology, nervous system

## Abstract

The Nile tilapia ( *Oreochromis niloticus* ) is a cichlid species native to Africa and is widely farmed in tropical and subtropical regions around the world, making it one of the most commercially important aquaculture species. Despite its significance, there is still a lack of detailed anatomical descriptions of its brain. This study aimed to provide a comprehensive anatomical and histological characterisation of the *O. niloticus* brain. Brain samples were collected from adult fish (*n* = 19) raised in a commercial aquaculture system in Brazil. The brain morphology of *O. niloticus* exhibits the general organisational pattern typical of teleosts, featuring two olfactory bulbs at the most cranial part of the brain, followed by prominent telencephalic lobes, a mesencephalon with a well‐developed pair of optic tectum, a ventrally located diencephalon with two large paired inferior lobes of the hypothalamus, a prominent cerebellum, and a large medulla oblongata. The gross and internal anatomy closely resembles that of other species within the same genus, such as *Oreochromis mossambicus* , as well as other cichlids and related teleosts, demonstrating a high degree of morphological similarity. In contrast, notable neuroanatomical differences are observed in more distantly related groups, such as Siluriformes and Gymnotiformes. Understanding the macroscopic and microscopic features of the brain can contribute to future studies in reproductive biology, behaviour, and systematics.

## 1 Introduction

Fishes represent the most species‐rich class of vertebrates (Kocher 2004). Owing to this diversity, there is considerable interspecific variation in the brain morphology of teleosts, both in external form and internal structure (Meek and Nieuwenhuys 1998). Nevertheless, despite the vast diversity of fish species, descriptive data on brain morphology remain limited to only a few groups.

The brain is a central organ responsible for sensory perception, motor functions, and behavioural responses (Kotrschal et al. 1998). Each brain region is associated with specific and essential functions and is interconnected with all systems of the organism (Abrahão and Shibatta 2015). Changes in brain morphology may be linked to variations in sensory orientation, cognitive abilities and motor skills (Ito et al. 2007).

The characterisation of brain structures in different fish species can be important for systematics and phylogenic studies, as various morphological features have been shown to be related to ecology, behaviour and evolution (Kotrschal et al. 1998; Abrahão et al. 2018). This anatomic knowledge also has practical applications in reflecting the specific ecological and behavioural demands required for each species, which can be helpful for fish reproduction and cultivation.

The anatomy of the fish brain provides crucial insights into the neural mechanisms underlying reproductive behaviours, stress responses and environmental adaptations essential for successful reproduction and aquaculture. Morphological studies can be directly linked to behavioural traits, supporting the development of strategies to enhance breeding success and optimise environmental conditions, thereby enabling targeted interventions to improve fish health in aquaculture settings.

Cichlid fishes are among the most successful taxa in vertebrate evolution (Simões et al. 2012). They represent a highly diverse group, comprising over 3000 species and exhibiting a wide range of phenotypic and behavioural variation, making them ideal for comparative analyses and valuable models for evolutionary studies (Kocher 2004). Although cichlid species serve as excellent models for comparative social neuroscience, data on their gross neuroanatomy remain limited (Huber et al. 1997; Gonzalez‐Voyer et al. 2009; Pollen et al. 2007), particularly concerning internal brain structures. Comprehensive anatomical descriptions exist for only a limited number of species, including *Astatotilapia burtoni* (Fernald and Shelton 1985; Burmeister et al. 2009; Maruska et al. 2012), *Cichlasoma dimerus* (Pandolfi et al. 2003) and *Oreochromis mossambicus* (Simões et al. 2012).

The Nile tilapia ( *O. niloticus* ) is a cichlid species of the Perciformes order, originally native to Africa. Its cultivation has gained significant importance in many tropical and subtropical regions worldwide, making it one of the most commercially farmed fish species (Pillay 1990; El‐Sayed 1999; FAO 2018). This success is attributed to several advantageous traits, including ease of reproduction, rapid growth, the feasibility of hormonal sex manipulation, omnivorous feeding habits, tolerance to low dissolved oxygen levels, disease resistance, and high‐quality meat that is widely appreciated for consumption and well suited for filleting (Simões et al. 2007).

Despite the species' intensive cultivation and economic importance, there are no published studies detailing the complete gross anatomy and histology of the brain of *O. niloticus* . Detailed brain morphology has been described only for *O. mossambicus* (Simões et al. 2012). Therefore, the present study aimed to describe the gross anatomy and detailed histological features of the brain of Nile tilapia ( *O. niloticus* ) from the tropical region of southeastern Brazil.

## 2 Material and Methods

### 2.1 Ethics for the Animal Use

The present research involved the use of fish specimens that were approved by the Ethics Committee for the Use of Animals—Comitê de Ética no Uso de Animais (CEUA) at the Federal University of Espírito Santo (protocol numbers: 19/2017, 11/2019).

### 2.2 Fish Samples

The adult *O. niloticus* specimens (*n* = 19) used in the present study were from Thai lineage obtained from a cultivation of the Laboratory of Nutrition and Production of Ornamental Fish (LNPEO/IFES) at the Espírito Santo Federal Institute (IFES) in Alegre, a municipality in the state of Espírito Santo, Brazil (Table 1). Fish were sampled from June 2017 to March 2019. The fish were anaesthetised with a benzocaine solution (100 mg/L), and necropsy was performed via cervical transection to remove the brain for subsequent morphological studies.

**TABLE 1:** Biometric measurements of *Oreochromis niloticus* specimens used in the present study, including total length (cm), total weight (g) and brain weight (g).

	Fish total length (cm)	Fish total weigh (g)	Brain weigh (g)
Minimum	7.2	6.9	0.04
Maximum	9.3	13.6	0.08
Average ± SD	8.21 ± 0.61	9.33 ± 2.20	0.06 ± 0.01

### 2.3 Analysis of Brain Anatomy

After removing the bones surrounding the lateral portions of the brain, the brain was exposed and immediately fixed with 4% buffered paraformaldehyde in phosphate buffer (pH 7.2) during dissection. This step was performed to preserve morphology and facilitate handling. The samples were carefully extracted from the skull, and brain images were captured using a camera attached to a Leica EZ4HD stereomicroscope, with magnifications ranging from 8× to 35×.

### 2.4 Analysis of Brain Histology

The samples were fixed using an immersion technique in 4% buffered paraformaldehyde in phosphate buffer (pH 7.2) for 24 h. Subsequently, they were dehydrated through increasing alcohol concentrations (70%, 80%, 90%, 100% (1) and 100% (2)), clarified in two baths of xylol, and embedded in paraffin wax. Cross‐sections were obtained starting from the most rostral extent of the olfactory bulb and continuing through the entire brain to the caudal spinal cord. Sections were not taken at regular intervals, but rather selected based on the appearance of novel anatomical structures. Sections of 0.4 μm were deparaffinised with xylol, rehydrated in decreasing alcohol concentrations (100%, 80%, 70%), stained with haematoxylin and eosin (H&E), and examined under a Leica ICC50HD light microscope. Images were captured using a camera attached to the Leica EZ4HD stereomicroscope, with magnifications ranging from 100× to 400×.

### 2.5 Statistics

Linear regression analysis was performed using brain weight, fish weight, and length data, with a 95% confidence interval. When necessary, data were transformed to meet the assumptions of the linear model (linearity, normality, homoscedasticity and leverage), employing a maximum likelihood estimation method (Venables and Ripley 2002). All statistical analyses were conducted using R version 4.1.0 (R Core Team 2020).

## 3 Results

**FIGURE 1:**
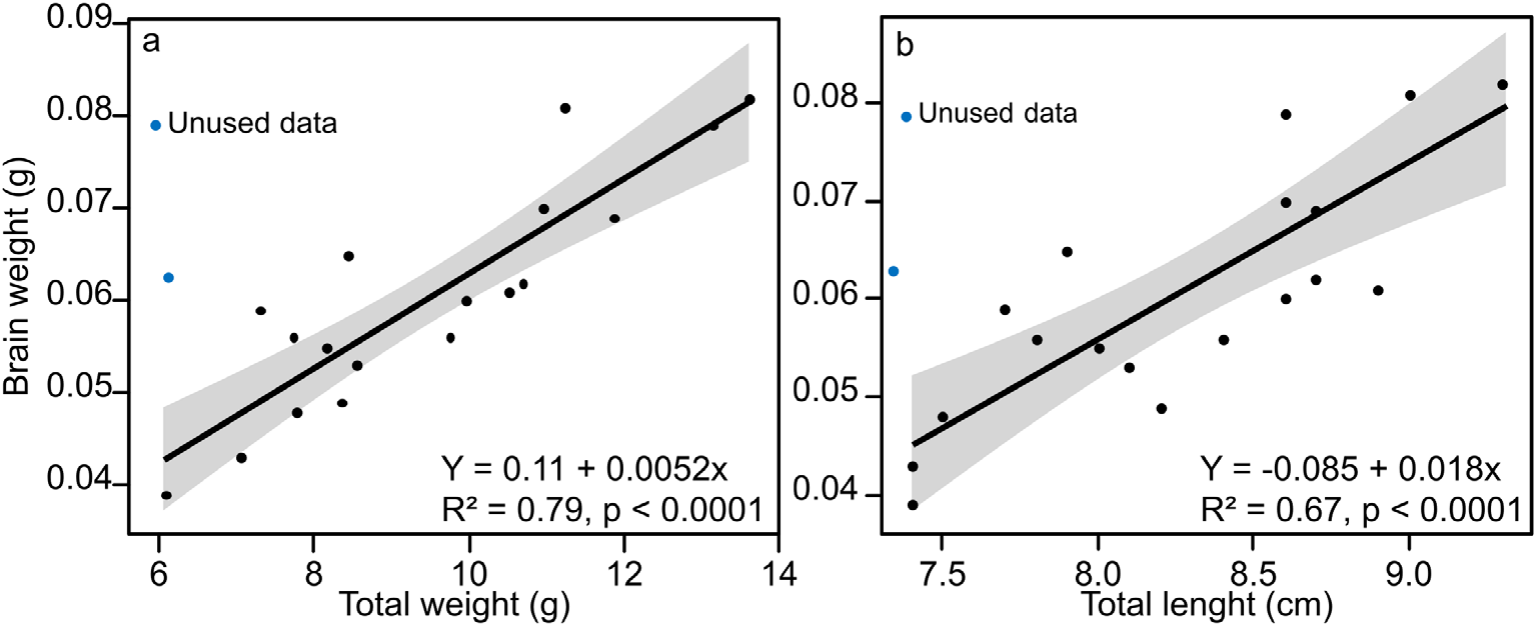
Linear regression between (a) total body weight and brain weight and (b) total body length and brain weight.

The brain of *O. niloticus* is light yellow to white in colour and shows a tendency to increase in weight with both body weight (*R* ^2^ = 0.79, *p* < 0.0001; Figure 1a) and body length (*R* ^2^ = 0.67, *p* < 0.0001; Figure 1b). The mean brain weight was 0.06 ± 0.01 g (Table 1), corresponding to approximately 0.7% of the total body weight.

**FIGURE 2:**
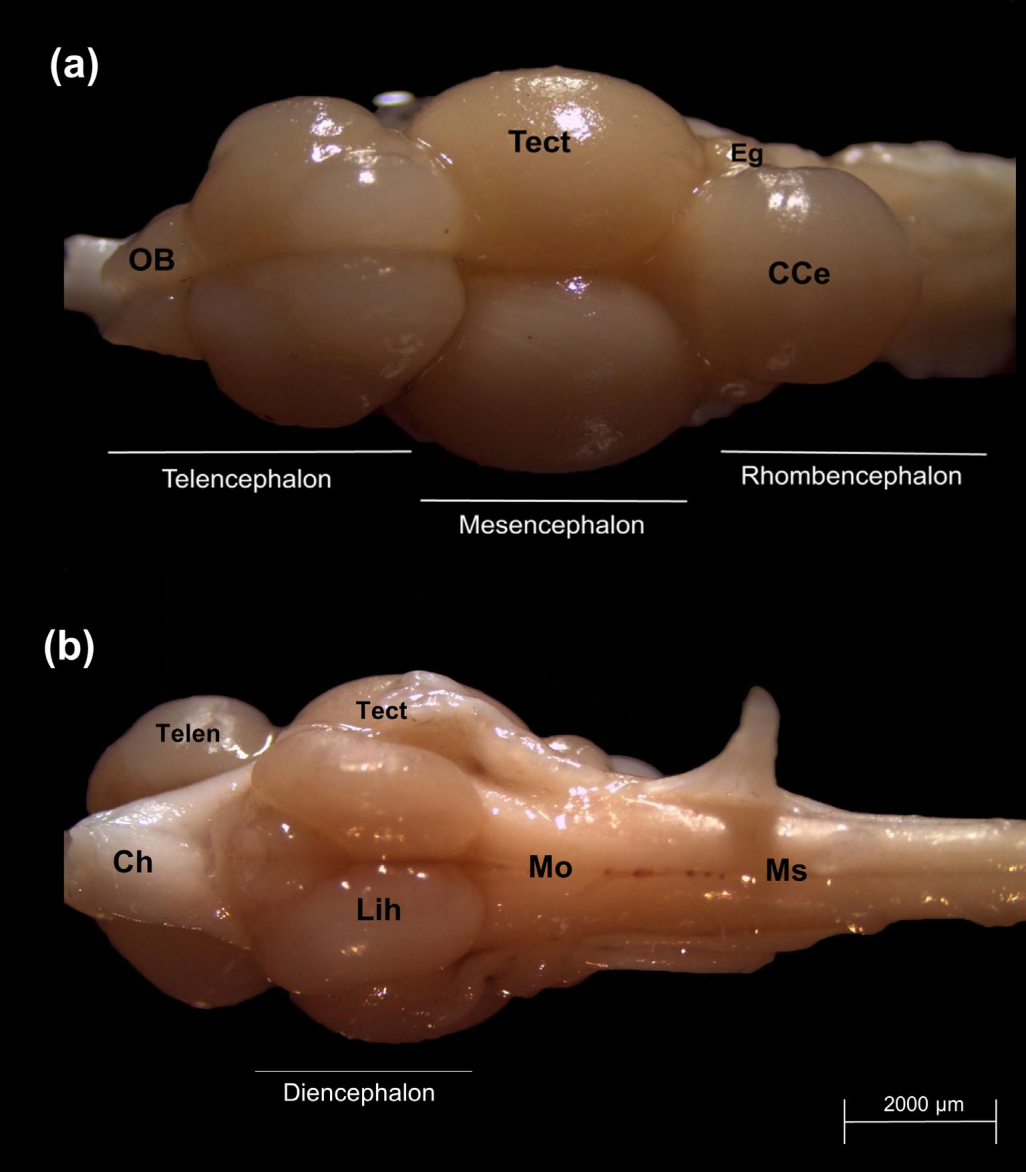
*Oreochromis niloticus* brain in (a) dorsal and (b) ventral view. CCe, Corpus cerebelli; Ch, Chiasma optic; Eg, Eminentia granularis; Lih, Inferior lobes of the hypothalamus; Mo, Medulla oblongata; Ms., Medulla spinalis; OB, Olfactory bulb; Tect, Optic tectum; Telen, Telencephalon.

**FIGURE 3:**
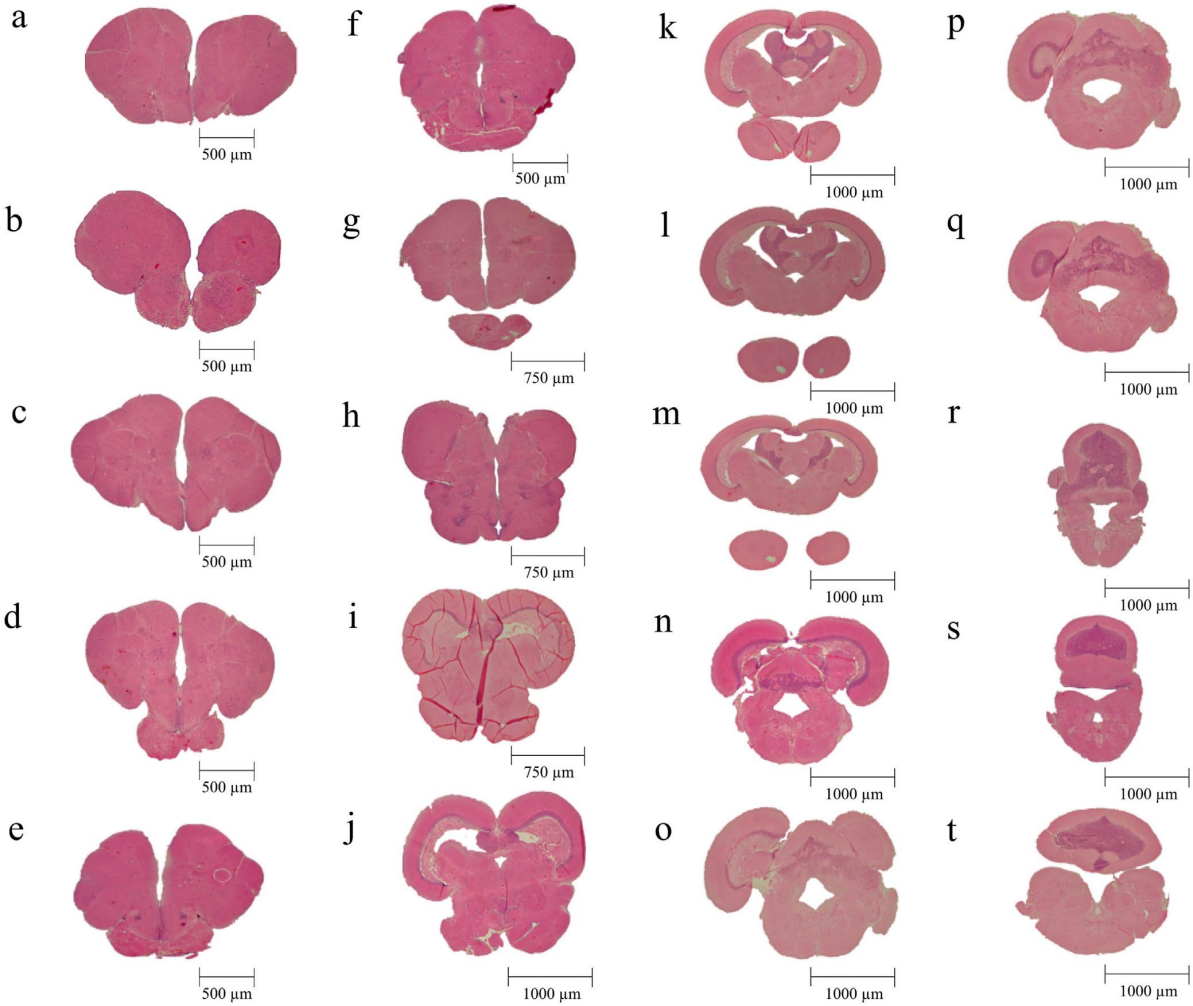
Neuroanatomy of the *Oreochromis niloticus* brain, showing different regions of the olfactory bulb (a–e), telencephalon (f–h), diencephalon (i–m), mesencephalon (n–o) and rhombencephalon (p–t).

The organ is slightly elongated and narrow, with its widest point located in the mid‐region, corresponding to the optic tectum (Tect) (Figure 2a,b). For descriptive purposes, the brain was divided into the olfactory bulbs (OB), telencephalon (Telen), diencephalon, mesencephalon and rhombencephalon (Figures 2 and 3, Table 2).

**TABLE 2:** Main structures observed in the brain regions of *Oreochromis niloticus* .

Brain region	Structures	Abbreviation	Figures
Telencephalon	Olfactory bulbs	OB	Figures 2a and 4a
	**Dorsal (Pallium)**		
	Dorsal zone of the dorsal telencephalon	Dd	Figure 4b
	Medial zone of the dorsal telencephalon	Dm	Figure 4b
	Lateral zone of the dorsal telencephalon	Dl	Figure 4b
	Central zone of the dorsal telencephalon	Dc	Figure 4b
	Posterior zone of the dorsal telencephalon	Dp	Figure 4b
	**Ventral (Subpallium)**		
	Dorsal zone of the ventral telencephalon	Vd	Figure 4b
	Ventral zone of the ventral ventral telencephalon	Vv	Figure 4b
	Post‐commissioning zone of the ventral telencephalon	Vp	Figure 4b
	Supracomissural zone of the ventral telencephalon	Vs	Figure 4b
Diencephalon	**Epithalamus**		
	Habenula	Hb	Figure 5a
	**Hypothalamus**		
	Lateral recess of the diencephalic ventricle	LR	Figure 5c
	Dorsal zone of the periventricular hypothalamus	HD	Figure 5c
	Inferior lobes of the hypothalamus	Lih	Figures 2b and 5c
	Lateral tuberal nucleus	NLT	Figure 5c
	**Thalamus**		
	Torus lateralis	TLa	Figure 5b
	Nucleus glomerulosus	nG	Figure 5b
Mesencephalon	Optic tectum	Tect	Figure 5a
	Torus semicircularis	TS	Figure 5c
	Torus longitudinalis	TL	Figure 5b,c
	Periventricular grey zone of the optic tectum	PGZ	Figure 5b,c
	**Truncus cerebelli**		
	Facial lobe	VIIL	Figure 6a
	Medial longitudinal fascicle	MLF	Figure 6a,b
Rhombencephalon	**Lobus vestibulolateralis**		
	Eminentia granularis	EG	Figures 2a and 6b
	**Corpus cerebelli**		
	Molecular layer of corpus cerebelli	CCe mol	Figure 6a,b
	Granular layer of corpus cerebelli	CCe gra	Figure 6a,b
	**Valvula cerebelli**		
	Granular layer of the lateral part of valvula cerebelli	Val gra	Figure 5c
	Molecular layer of the medial part of valvula cerebelli	Vam mol	Figure 5c
	Molecular layer of the lateral part of valvula cerebelli	Val mol	Figure 5c

### 3.1 Telencephalon

The olfactory bulbs are a pair of structures located at the most rostral part of the brain, representing the first component of the telencephalon (Figure 2a). This region is followed by a dorsally prominent pair of solid telencephalic lobes (Figure 2a), which extend posteriorly to the optic tectum (Figure 2b).

**FIGURE 4:**
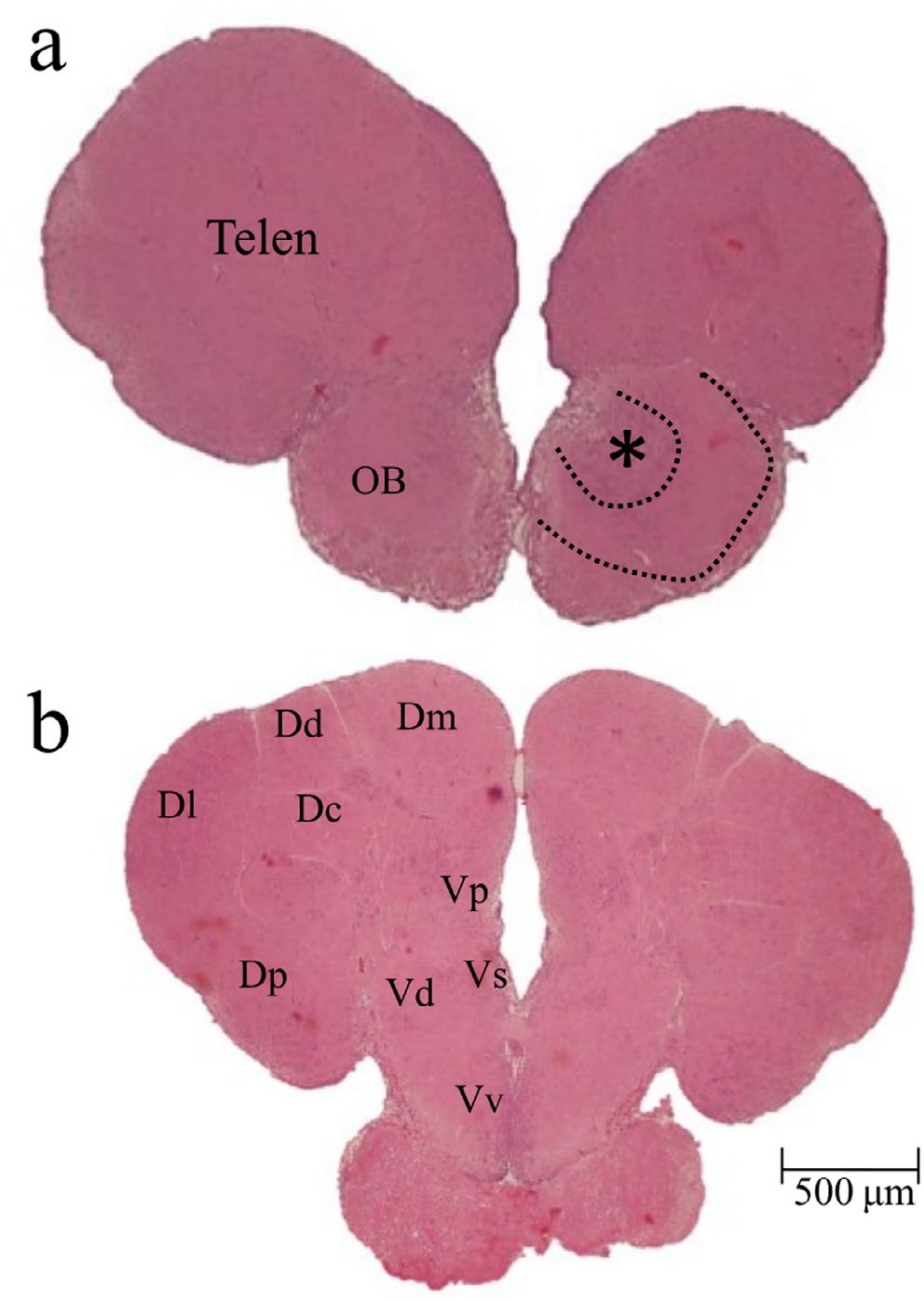
Photomicrographs of cross sections of the *Oreochromis niloticus* telencephalon. OB, olfactory bulb; OT, optic tractus; Telen, telencephalon. Dorsal telencephalon (pallium) includes Dc, central; Dd, dorsal; Dl, lateral; Dm, medial; Dp, posterior regions. Ventral telencephalon (subpallium) includes Vd, dorsal; Vp, post‐commissioning; Vs, supracomissural regions; Vv, ventral. Lines indicate the concentric layer organisation of the olfactory bulbs, and the asterisk marks the glomerular region.

Histological examination revealed that the olfactory bulbs are organised into external concentric layers (Figure 4a, line), formed by olfactory nerve fibres running toward the telencephalic hemispheres, and an internal glomerular layer, characterised by glomerular masses (Figure 4a, asterisk).

The telencephalon is divided into two distinct regions: the dorsal part (pallium, P) and the ventral part (subpallium, SP) (Figure 4b). Histologically, it is possible to observe subdivisions within both regions (Figure 4b; Table 2). The dorsal region (pallium) is characterised by the medial (Dm), dorsal (Dd), lateral (Dl), central (Dc) and posterior (Dp) subdivisions (Figure 4b). The ventral region (subpallium) is composed of the dorsal (Vd), ventral (Vv), posterior (Vp) and supracommissural (Vs) subdivisions (Figure 4b). Both dorsal and ventral regions receive direct input from the olfactory bulb.

### 3.2 Diencephalon

The diencephalon is located in the most ventral region of the brain, situated between the telencephalon and mesencephalon (Figure 2b). In *O. niloticus* , the diencephalon comprises three main subdivisions: the thalamus, epithalamus and hypothalamus.

**FIGURE 5:**
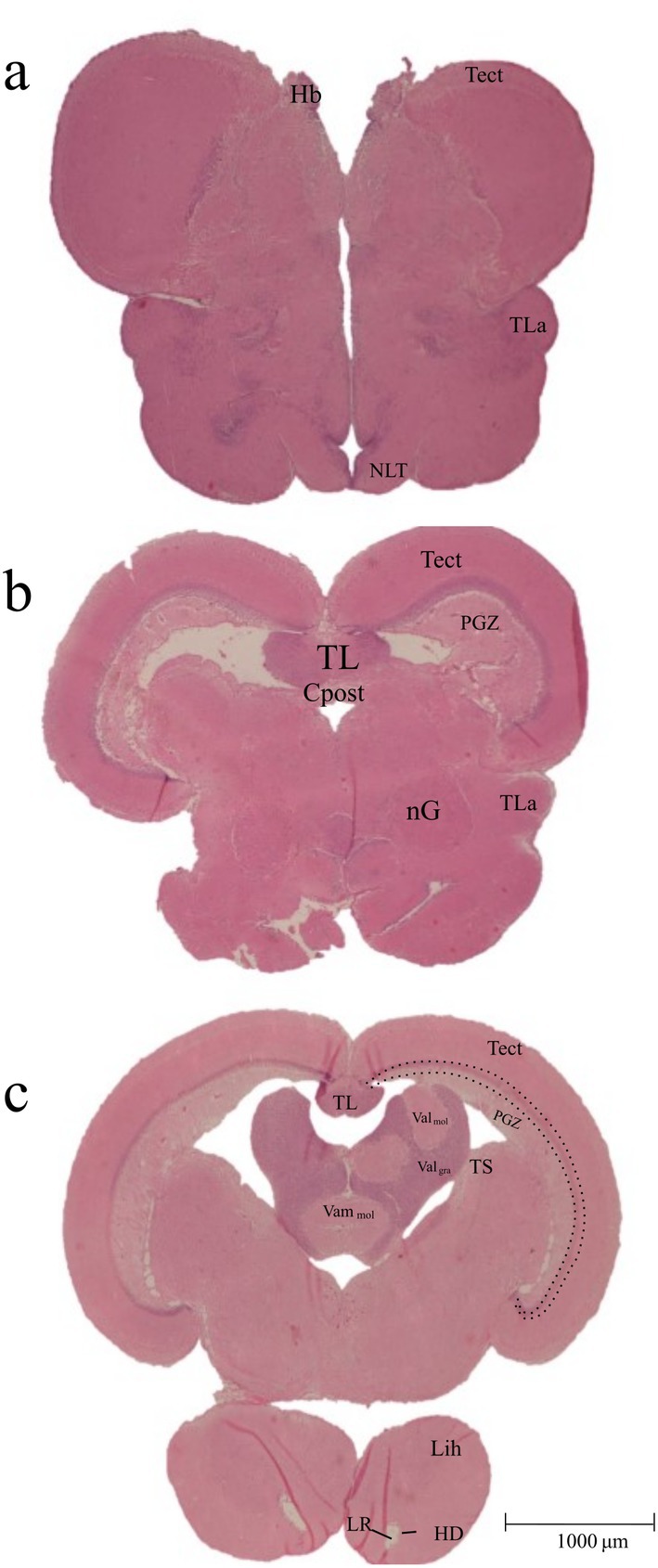
Photomicrographs of cross sections of the *Oreochromis niloticus* diencephalon. Hb, Habenula; HD, Dorsal zone of the periventricular hypothalamus; Lih, Inferior lobes of the hypothalamus; LR, Lateral gap of the diencephalic ventricle; nG, Nucleus glomerulosus; NLT, Lateral tuberal nucleus; PGZ, Periventricular grey zone of the optic tectum; Tect, Optic tectum; TL, Torus longitudinalis; TLa, Torus lateralis; TS, Torus semicircularis; Valgra, Granular layer of the lateral part of the valvula cerebelli; Valmol, Molecular layer of the lateral part of the valvula cerebelli; Vammol, Molecular layer of the medial part of the valvula cerebelli. Dotted lines indicate the granular cell layer of the optic tectum.

The thalamus is a small structure located on the lateral surface of the brain, including the torus lateralis (TLa) region (Figure 5a) and the nucleus glomerulosus (nG) (Figure 5b). The nucleus glomerulosus is a concentrically layered nucleus that sends efferent projections to the inferior lobes of the hypothalamus (Lih) (Figure 5b).

The epithalamus is composed of the habenula (Hb), the habenular commissure, and the pineal gland. The habenula lacks evident symmetry but is prominently located in the internal lateral region of the optic tectum (Figure 5a).

The hypothalamus can be subdivided into three major regions: the periventricular region, the tuberal region (which includes the lateral tuberal nucleus—NLT) (Figure 5a), and the inferior lobes of the hypothalamus (Figure 2b). The inferior lobes form a prominent paired structure with a cylindrical to semi‐circular shape, consisting of medially connected lobes that occupy the largest portion of the diencephalon, located in the most posterior part of the hypothalamus (Figure 2b). The dorsal zone of the hypothalamus is the most visible portion and includes both the inferior lobes and the lateral gap of the diencephalic ventricle (LR), which is surrounded by the dorsal zone of the periventricular hypothalamus (HD) (Figure 5c).

### 3.3 Mesencephalon

The mesencephalon, or midbrain, is located between the telencephalon and rhombencephalon. It consists of a well‐developed dorsal pair of optic tectum (Figure 2a) and a ventral mesencephalic tegmentum, which is entirely covered by the inferior lobes of the hypothalamus. Together, these two symmetrical, rounded structures occupy approximately one‐third of the total brain length (Figure 2a).

The mesencephalon is organised in distinct layers, which, from outer to inner regions, include the optic tectum, followed by a densely stained granular cell layer (Figure 5c, lines), and the periventricular grey zone (PGZ) (Figure 5b,c). In addition to the optic tectum, the mesencephalon also contains the torus longitudinalis (TL) and the torus semicircularis (TS) (Figure 5b). These regions were only visible histologically at the borders of the ventricles (Figure 5c).

The torus longitudinalis is a paired midline structure of the dorsal mesencephalon that protrudes into the ventricle in close proximity to the valvula of the cerebellum (VCe) (Figure 5b,c). These structures form a functional visual connection between the cerebellum and the optic tectum. The torus semicircularis is another potentially important structure associated with the optic tectum, projecting axons to both tectal lobes.

**FIGURE 6:**
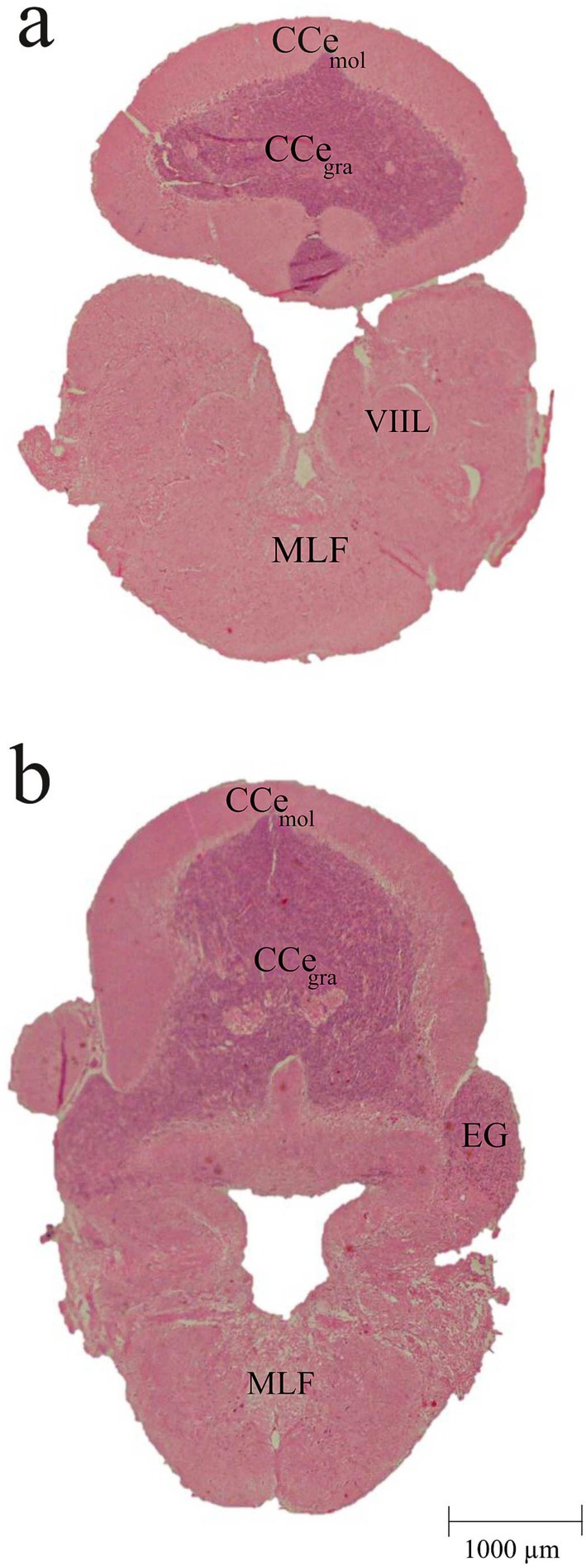
Photomicrographs of cross sections of the *Oreochromis niloticus* rhombencephalon. CCe gra, granular layer of the corpus cerebelli; CCe mol, molecular layer of the corpus cerebelli; Eg, eminentia granularis; MLF, medial longitudinal fasciculus; VIIL, facial lobe.

In addition, the truncus cerebelli is a notable region located between the mesencephalon and rhombencephalon. This area contains the facial lobe (VIIL) (Figure 6a) and the medial longitudinal fasciculus (MLF) (Figure 6a,b).

### 3.4 Rhombencephalon

The rhombencephalon is the most posterior part of the brain, being divided into medulla oblongata (Mo) and cerebellar complex or cerebellum (Figure 2a). The cerebellar complex comprises three principal subdivisions: the corpus cerebelli (CCe), the valvula cerebelli and the lobus vestibulolateralis.

The corpus cerebelli (CCe) is the major component of the rhombencephalon and, in this species, has a spherical shape, located dorsally to the medulla oblongata (Mo) (Figure 2a). It is divided into an external molecular layer (CCe mol) and an internal granular layer (CCe gra) (Figure 6a,b).

In *O. niloticus* , the valvula of the cerebellum consists of lateral (Valmol, Valgra) and medial (Vammol) regions (Figure 5c). It is a protrusion into the mesencephalic ventricle that is found only in actinopterygians.

The lobus vestibulolateralis is composed of the eminentia granularis (Eg) and the lobus caudalis (Figure 6b), and is closely associated with the vestibular and lateral line systems. The eminentia granularis appears as two lateral flaps located anterior to the cerebellar complex, seemingly continuous with the granular layer of the corpus cerebelli (Figures 2a and 6b).

## 4 Discussion

The observations of the present study contribute to the understanding of *O. niloticus* brain morphology, as detailed anatomical and histological features are demonstrated for the first time in this species. The knowledge of the macroscopic and microscopic characteristics of the brain may contribute to further systematic, reproduction, behavioural and physiological studies.

In the present study, the *Oreochromis niloticus* brain weight was 0.7% of the body weight, with the positive correlation between these variables. Differences in relative sizes and morphology of fish brains are related to phylogenetic development and may manifest in the complexity and compactness of the organ (Kotrschal and Kotrschal 2020). Some primate fish (as coelacanth) weigh considerably less than 0.1 of total body weight, cyprinid minnows showed relative brain weight with at least twice that, while mormyrids of Africa showed the largest relative size, reaching more than 1% of body weight (Nieuwenhuys 1982). These changes are often due to relative differences in the development of the special senses (sight, hearing, touch, smell, taste and mechanosensory and electroctrosensory lateral line) (Meek and Nieuwenhuys 1998).

The gross morphology of the *O. niloticus* brain resembles that observed in other teleosts, featuring two olfactory bulbs at the most cranial part of the brain, followed by prominent telencephalic lobes, a mesencephalon with a well‐developed pair of optic tectum, a ventrally located diencephalon with two large paired inferior lobes of the hypothalamus, a prominent cerebellum and a large medulla oblongata.

The gross anatomy of the *O. niloticus* brain is similar in appearance, organisation, and size to that of *O. mossambicus* (Simões et al. 2012), other cichlid species (Huber et al. 1997), and closely related teleosts of the order Cyprinodontiformes, such as *Nothobranchius furzeri* (D'Angelo 2013) and *Austrolebias* (Fernández et al. 2011), showing a high degree of similarity. The main differences lie in the size of the olfactory bulbs and the relative proportions of the telencephalon and optic tectum. The olfactory bulbs are smaller in *N. furzeri* (D'Angelo 2013) and *Austrolebias* (Fernández et al. 2011) than those observed in the *Oreochromis* genus (Simões et al. 2012; present study). In *Austrolebias*, the telencephalon is similar in size to the tectal lobes (Fernández et al. 2011), whereas in *Oreochromis*, the telencephalon is comparatively smaller (Simões et al. 2012; present study).

Significant and relevant differences in neuroanatomical architecture are observed in more distantly related groups such as Siluriformes—for example, *Pseudopimelodus bufonius* (Abrahão and Shibatta 2015) and other catfish species (Abrahão et al. 2018)—as well as in Gymnotiformes (Alberts 2001) and Characiformes (Pereira and Castro 2016). These groups display reduced olfactory bulbs, tectal lobes (particularly in Siluriformes) and inferior lobes of the hypothalamus, along with a prominent cerebellum (especially in Siluriformes and Gymnotiformes) (Abrahão and Shibatta 2015; Abrahão et al. 2018; Alberts 2001; Pereira and Castro 2016).

The olfactory bulb, the most anterior structure of the brain, serves as the primary centre for processing olfactory information (Nieuwenhuys 1982). It is situated on the ventral surface of the telencephalon, where the olfactory tract nerves converge, and its size varies according to the olfactory capabilities of different species. The size of the olfactory bulbs reflects the development of the olfactory system (Nieuwenhuys 1982). In *O. niloticus* , the olfactory bulbs are similar in size and morphology to those of *O. mossambicus* (Simões et al. 2012), but smaller than in closely related teleost species from the Cyprinodontiformes order, such as *N. furzeri* (D'Angelo 2013) and *Austrolebias* (Fernández et al. 2011). Conversely, *O. niloticus* olfactory bulbs are relatively larger than those observed in more distantly related teleost groups, including Siluriformes (Abrahão and Shibatta 2015; Abrahão et al. 2018) and Gymnotiformes (Alberts 2001). The size of the olfactory bulbs also appears to be influenced by environmental factors, feeding habits, and microhabitat use. For example, African cichlids with piscivorous diets tend to have larger olfactory bulbs compared to insectivorous and zooplanktivorous species (Huber et al. 1997).

It is generally accepted that the telencephalon plays a key role in processing visual inputs. The majority of optic fibres terminate in the optic tectum, making this region the primary visual centre of the brain (Northmore 2011). Additionally, the telencephalon is involved in transmitting olfactory information and contributes to behaviours such as feeding, defence, learning, aggression and reproduction (Northcutt 1983; Wullimann 1997). The telencephalic morphology observed in *O. niloticus* is similar to that described in other Perciformes species, such as *O. mossambicus* (Simões et al. 2012) and *Sparus aurata* (Muñoz‐Cueto 2001), as well as in closely related teleosts like *N. furzeri* from the Cyprinodontiformes order (D'Angelo 2013). Beyond evolutionary relationships, telencephalon size is also influenced by environmental conditions and species‐specific habits; for example, African cichlids from shallow waters tend to have larger telencephalons compared to those from deeper waters (Huber et al. 1997). Thus, visual habitat conditions significantly affect brain morphology, as factors like water turbidity and depth have a pronounced impact on the size of visual brain structures (Huber et al. 1997).

In *O. niloticus* , the telencephalon is divided into dorsal (pallium) and ventral (subpallium) regions, which are homologous to those of tetrapods and mammals (Meek and Nieuwenhuys 1998; Braford 1995). Similar subdivisions of the pallium and subpallium have also been observed in the congeneric species *O. mossambicus* (Simões et al. 2012) and in closely related teleost species such as *Austrolebias* (Fernández et al. 2011). The dorsal telencephalon (pallium) of both tilapiine species is organised in distinct areas as lateral (Dl), medial (Dm), central (Dc), dorsal (Dd) and posterior zone (Dp). However, the subdivisions within each of these regions do not consistently correspond between the two species. Moreover, it remains unclear to what extent these differences in nomenclature reflect actual cytoarchitectural variations or simply divergent interpretations among researchers. Future studies employing genetic markers may help resolve these inconsistencies (Simões et al. 2012).

The diencephalon, which consists of the epithalamus, thalamus, and hypothalamus, is considered the most complex region of the teleost brain (Nieuwenhuys 1982). The hypothalamus is subdivided into three major regions: the periventricular zone, the tuberal region, and the inferior lobes. The inferior lobes occupy the largest portion of the diencephalon and receive sensory input from gustatory centres, the suprachiasmatic nuclei, and the ventral region of the telencephalon (Finger and Kanwal 1992). In some Perciformes species, the hypothalamus is surrounded by the saccus vasculosus, a sensory organ involved in detecting photoperiodic signals (Nakane et al. 2013), as in *Dolloidraco longedorsalis* from Antarctica (Eastman and Lannoo 2003). However, the saccus vasculosus has not been described in *O. niloticus* (present study) or *O. mossambicus* (Simões et al. 2012).

Together, the thalamic and hypothalamic regions of the diencephalon receive fibres from the optic tectum, cerebellum, and various brainstem centres. Additionally, the diencephalon contains the nucleus glomerulosus, a prominent, concentrically layered nucleus that sends efferent projections to the inferior lobes of the hypothalamus (Muñoz‐Cueto 2001). This structure has also been observed in *O. mossambicus* (Simões et al. 2012) and in closely related teleost orders such as Cyprinodontiformes, including *N. furzeri* (D'Angelo 2013).

The mesencephalon consists of two main regions: the ventral tegmentum and a pair of well‐developed dorsal optic tectum. In fish, as in most other vertebrate groups, the primary function of the mesencephalon is visual processing, although it also supports somatosensory, auditory, lateral line, and electrosensory modalities (Northmore 2011), resulting in the generation and coordination of motor responses (Meek and Nieuwenhuys 1998). The mesencephalon connects with motor centres, enabling fish to respond rapidly and appropriately to stimuli, whether for prey capture or the avoidance of objects and predators (Northmore 2011).

The size of the optic lobes varies among species and is particularly large in visually oriented fishes (Nieuwenhuys 1982). *O. niloticus* exhibits a large optic tectum, similar to those observed in other cichlid species (Simões et al. 2012; Huber et al. 1997), in closely related teleost orders such as Cyprinodontiformes (D'Angelo 2013; Fernández et al. 2011), and even in more distantly related groups, such as Characiformes (Pereira and Castro 2016) and Cypriniformes (Wullimann et al. 1996). In contrast, smaller tectal lobes are observed in Siluriformes (Abrahão and Shibatta 2015; Abrahão et al. 2018) and Gymnotiformes (Alberts 2001). The size of the tectal lobes is also influenced by species' habits and environmental conditions; for instance, African cichlids from deeper habitats tend to have smaller optic tectum, whereas pelagic species exhibit larger ones (Huber et al. 1997).

The rhombencephalon is the most posterior part of the brain and is divided into the medulla oblongata and the cerebellar complex (cerebellum). The cerebellum plays a key role in coordinating movement, regulating muscle tone, and maintaining posture and balance (Paulin 1993). Its size and complexity vary across species, ranging from a relatively simple structure in ancestral or sedentary benthic fishes to a well‐developed structure in modern teleosts (Kotrschal and Kotrschal 2020). In *O. niloticus* (present study), the cerebellum is highly developed and shows homology with that of other cichlid species (Simões et al. 2012; Huber et al. 1997), as well as with closely related teleost orders such as Cyprinodontiformes (D'Angelo 2013; Fernández et al. 2011), and even with more distantly related groups such as Characiformes (Pereira and Castro 2016) and Cypriniformes (Wullimann et al. 1996). The cerebellum is especially prominent in more distantly related teleosts such as Gymnotidae (Alberts 2001) and Siluriformes (Abrahão and Shibatta 2015; Abrahão et al. 2018). Environmental conditions and species‐specific behaviours also influence cerebellar development. For example, piscivorous African cichlids and other species that hunt motile prey tend to have larger optic tectum and cerebellum, suggesting that enhanced ability to prey detection together with improved motor coordination for pursuit and capture (Huber et al. 1997).

One of the main challenges in descriptive neuroanatomical studies is the limited number of publications available for certain species, primarily due to the vast diversity of fish. Moreover, many existing studies lack detailed descriptions of internal brain structures. Consequently, there is a continued need for comprehensive and standardised descriptions of fish brain anatomy. This study provides a detailed anatomical and histological description of the brain of *O. niloticus* , offering a valuable reference for future morphological and functional research on this species.

## 5 Conclusion

This study provides a detailed anatomical and histological description of the brain of *O. niloticus* . The gross and internal anatomy closely resembles that of other species within the same genus, such as *Oreochromis mossambicus* , as well as other cichlids and related teleosts, demonstrating a high degree of morphological similarity. In contrast, notable neuroanatomical differences are observed in more distantly related groups, such as Siluriformes and Gymnotiformes. The knowledge from specific brain regions is relevant for tilapiine cichlids, especially due to their application in aquaculture. Understanding the brain structure and connectivity of these areas can be developed methods to stimulate or modulate reproductive cycles. Therefore, the results from the present study can contribute to further research in reproductive biology, behaviour, and systematics.

## Conflicts of Interest

The authors declare no conflicts of interest.

## Data Availability

Data sharing is not applicable to this article as no datasets were generated or analysed during the current study.
